# Adjuvant inhibition of iAPC function in the tumor microenvironment promotes therapeutic immunity in the setting of vaccination-induced T cell anti-tumor response

**DOI:** 10.1186/2051-1426-3-S2-P393

**Published:** 2015-11-04

**Authors:** Joseph Antonios, Horacio Soto, Joey Orpilla, Namjo Shin, Richard Everson, Linda Liau, Robert Prins

**Affiliations:** 1 UCLA Neurosurgery, Los Angeles, CA, USA

## 

Glioma tumor lysate-pulsed dendritic cell (DC) vaccination is an effective treatment modality. However, cure rates in the established tumor setting are not therapeutically significant in our preclinical models. We inferred that immunosuppressive antigen presenting cells (iAPCs) present in the tumor environment acting via the PD-1/PD-L1 mechanism mediated immune suppression in malignant glioma. To test this hypothesis in our *in vivo* preclinical model, mice intracranially implanted with GL261 gliomas were treated with DC vaccination +/- murine anti-PD-1 mAb (RMP1-14, Bioxcell) blockade or a CNS-penetrant small molecule inhibitor of CSF-1R (PLX-3397, Plexxikon) and overall survival was quantified. We then harvested and characterized the intratumoral cellular infiltrate and demonstrated the presence of a PD-L1^+^ CD11b^+^ CSF1r^+^ inhibitory antigen-presenting cell (iAPC) population. Gene expression profiles of harvested iAPCs were assessed using the novel Nanostring nCounter analysis system. We found that treatment with DC vaccination and adjuvant PD-1 mAb blockade and PLX-3397 induced a highly significant therapeutic benefit to animals bearing well-established i.c. gliomas (and the inhibition of iAPC negative regulatory function.

